# 3D Printed Skull Cap and Benchtop Fabricated Microwire-Based Microelectrode Array for Custom Rat Brain Recordings

**DOI:** 10.3390/bioengineering9100550

**Published:** 2022-10-14

**Authors:** Dongyang Yi, Jeremiah P. Hartner, Brian S. Ung, Harrison L. Zhu, Brendon O. Watson, Lei Chen

**Affiliations:** 1 Department of Mechanical Engineering, University of Massachusetts Lowell, Lowell, MA 01854, USA; 2 Department of Psychiatry, University of Michigan, Ann Arbor, MI 48109, USA

**Keywords:** microwire microelectrode array, skull cap, multi-region electrophysiological recording, 3D printing, custom design, normal-behaving rat brain recording

## Abstract

Microwire microelectrode arrays (MEAs) have been a popular low-cost tool for chronic electrophysiological recordings and are an inexpensive means to record the electrical dynamics crucial to brain function. However, both the fabrication and implantation procedures for multi-MEAs on a single rodent are time-consuming and the accuracy and quality are highly manual skill-dependent. To address the fabrication and implantation challenges for microwire MEAs, (1) a computer-aided designed and 3D printed skull cap for the pre-determined implantation locations of each MEA and (2) a benchtop fabrication approach for low-cost custom microwire MEAs were developed. A proof-of-concept design of a 32-channel 4-MEA (8-wire each) recording system was prototyped and tested through Sprague Dawley rat recordings. The skull cap design, based on the CT-scan of a single rat conforms well with multiple Sprague Dawley rats of various sizes, ages, and weight with a minimal bregma alignment error (A/P axis standard error of the mean = 0.25 mm, M/L axis standard error of the mean = 0.07 mm, n = 6). The prototyped 32-channel system was able to record the spiking activities over five months. The developed benchtop fabrication method and the 3D printed skull cap implantation platform would enable neuroscience groups to conduct in-house design, fabrication, and implantation of customizable microwire MEAs at a lower cost than the current commercial options and experience a shorter lead time for the design modifications and iterations.

## 1. Introduction

The electrophysiological recording of single neuron activities is critical for understanding the brain functioning mechanisms [1,2]. To gain further insight into the dynamic neural mechanisms and interactions across the different brain regions, the simultaneous chronic recording from multiple locations at a high temporal resolution in freely behaving animals is needed [3,4,5]. Single neuron activities, in the form of action potentials, last about 1 ms, the synaptic delays range from 1–10 ms [6,7], and the neural assemblies’ function at tens of milliseconds [8]. Such action potential is only distinguishable within an approximately 150 μm radius [9], making it necessary to implant microelectrode arrays (MEAs) in the brain and to place the recording sites right next to neurons. In comparison, the electrophysiology methods, such as electroencephalography (EEG), magnetoencephalography (MEG), and functional magnetic resonance imaging (fMRI) lack the spatial and/or temporal resolution to record the single neuron activities [10,11]. The implantation of MEAs has evolved for decades and has been proven to be a powerful tool to provide high temporal resolution recordings of action potentials [12,13,14,15]. Despite the emerging development of the silicon based MEAs in recent decades, microwire MEAs, first developed in the 1950s [16], are still a popular choice for neuroscientists due to their low cost [17], high temporal resolution, and great chronic recording performance, thanks to their high flexibility and small size [18,19].

Since the brain is composed of multiple interacting regions, multiple MEAs implanted across regions can reveal fast timescale electrical dynamics crucial to brain function. The current approaches for multiple microwire MEA recordings in rodents rely on the repeated single implant craniotomes and other tedious stereotactic-based surgical procedures that are not automated, nor do they take advantage of modern mechanical tools. Specifically, in typical surgeries, the implant locations are individually determined within surgery and a separate craniotomy and durotomy are performed at each location [20,21]. During each implantation, the insertion depth (Dorsal/Ventral) is also controlled by manual operation and measurement through the stereotactic device or linear stage [22,23]. Temporary apparatus, such as thin metal bars and 3D printed custom fixtures, are used to hold the MEA in place until the cured dental cement fixes the electrode at its final position [24,25]. As a result, a three-dimensional positioning accuracy and repeatability of each implantation position are highly dependent on surgical skill and practice. The manual manipulation and alignment of multiple MEAs within a small area of the animal brain surface is labor-intensive and easily leads to complex surgeries lasting many hours. New approaches are needed to simplify the surgery procedure and increase the accuracy and repeatability of the multi-MEA insertion locations. Moving towards to the use of computer-based designs can open the door to later future automation and higher throughput. But an initial movement away from all manual approaches is needed first.

Fabricating MEAs themselves is also a highly manual skill-dependent process. The general fabrication steps involve the electrode alignment which counts on manual work with grids and hands-on small wire soldering [26,27,28], and both procedures are time-consuming. Such a labor-intensive fabrication process has driven many neuroscientists toward commercially available microwire MEAs [29,30]. These arrays, while ready off-the-shelf, require recording experiments to be designed around the available configurations [22,31] and would result in higher costs and a longer lead time for customized MEA geometry needs.

To enable rodent neuroscience groups to design, fabricate, and implant custom microwire MEAs in house, this study developed (1) a digitally designed and 3D printed custom skull cap with predetermined insertion locations and depths for accurate but simplified multi-region recordings and (2) a benchtop fabrication approach for low-cost, highly customizable, and in-house made microwire-based MEAs using easily accessible materials and tools with minimal training needed. To demonstrate the feasibility and reliability of the developed methodology to provide in-vivo rat brain electrophysiological recordings, a proof-of-concept surgery was planned and conducted to implant four 8-channel planar microelectrode arrays of 50 μm diameter tungsten microwires and 1 mm pitch to record from the cortical areas of a Sprague Dawley rat brain. This 32-channel MEA configuration allows us to span broadly along the anterior-posterior axis of the rat brain and showcase the surgery simplification enabled by the 3D printed custom skull cap.

## 2. Materials and Methods

In this section, the design and fabrication approach for our 32-channel 4-MEA recording system is elaborated as a demonstration of our 3D printed skull cap and benchtop microwire MEA fabrication method. The generalization strategy to move beyond this application-specific design is described at the end of each subsection.

### 2.1. Recording Apparatus Overview and the Printed Circuit Boards Design

**Figure 1:**
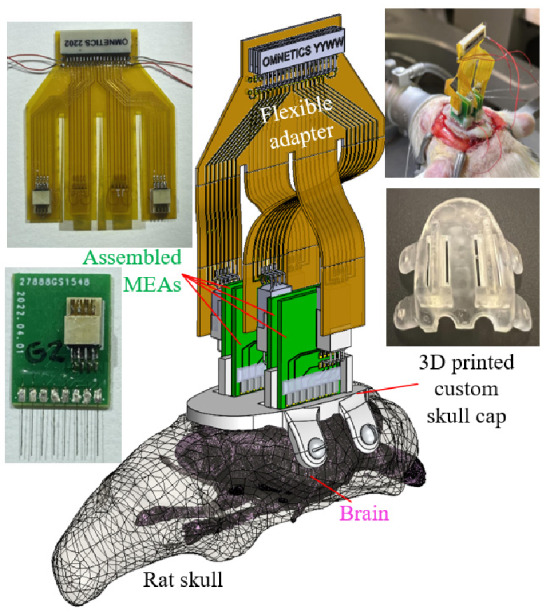
Major components overview of the recording apparatus.

Figure 1 shows the overview of the recording apparatus proposed for the multiple MEA recording paradigm. Three major components were needed to complete the apparatus: (1) a digitally designed and 3D printed skull cap with structures to guide and position each MEA implantation (elaborated in Section 2.2), (2) the assembled microwire-based MEAs with custom configurations (to be described in Section 2.3), and (3) the corresponding printed circuit boards (PCBs) for both the electrical connection of microwires in each array and the connection of multiple arrays to the proposed headstage.

**Figure 2:**
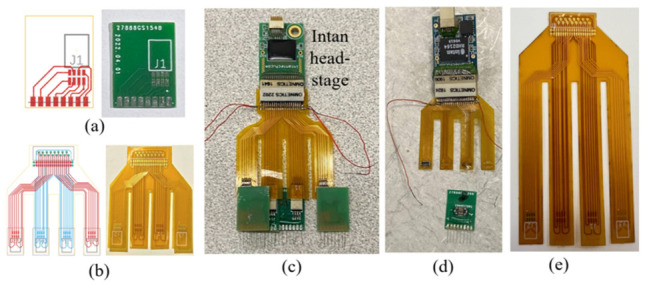
Digital design and the fabricated pieces of (**a**) the microwire MEA PCB and (**b**) the flexible adapter PCB and (**c**) the final assembled electronics with the headstage. The same design concept could also be implemented with other preferred (**d**) connectors and/or (**e**) configurations.

In this demonstration study, the 32-channel recording headstage (RHD 32 by Intan Technologies, Los Angeles, CA, USA) determined the end female connector (A79025 by Omnetics Connector Corporation, Minneapolis, MN, USA) that the developed electrical connection system would be connected to. To provide the connections between each of the 32 microwires to one of the pins of the headstage connector, a two-stage PCB system was proposed and developed, as shown in Figure 2. The first stage (Figure 2a) was a rigid PCB connecting eight (8) microwires in an array to an eight-pin connector (A79613-001 by Omnetics Connector Corporation, Minneapolis, MN, USA). The eight gold pads towards the bottom edge, matched the microwire array pitch (1 mm) and would connect to the individual microwires. The other eight connection pads were patterned based on the eight-pin Omnetics connector. Note that the connector was placed off-center on the PCB, which was meant to maximize the rat skull space usage by staggering the PCBs together (to be described in Section 2.3). The second stage of the PCB system (Figure 2b) was a flexible PCB adapter connecting four eight-pin connectors from the MEA arrays to a male 36 position connector (A79024-001 by Omnetics Connector Corporation, Minneapolis, MN, USA) paired with the 32-channel headstage. Moreover, the 32 microwire connections and the remaining four pins were used for ground and reference wires, as shown in the assembled electronics in Figure 2c. To minimize the flexible adapter PCB size, a dual-side circuit board was designed and fabricated with connections to two MEAs on each side. Four long flexible “legs” of the PCB enabled the flexible connection after the MEA implantation, to be shown in Section 2.4. Both PCBs were designed digitally (EAGLE by Autodesk Inc., San Rafael, CA, USA) and outsourced for their fabrication (PCBminions Inc., Princeton, NJ, USA). The rigid PCB was fabricated with a FR-4 glass-reinforced epoxy laminate material, 0.6 mm board thickness, hot air solder leveling surface finish, and 1 oz finished copper. The 2-layer adapter PCB in this study was made of 0.5 mil thick polyimide for optimized flexibility.

The PCB designs and parameters shown in this proof-of-concept trial were tailored, based on the specific recording species and the task in this study. The same concept of a two-stage connection system composed of a planar rigid and flexible PCBs could be implemented with different MEA pitch sizes, various total channel counts and distribution (determining wire number in each MEA), the preferred types of connectors (prototype based on Hirose^TM^ connectors shown in Figure 2d), and custom flexible adapter configurations (adapter PCB with longer “feet” shown in Figure 2e) for specific species and desired recording locations.

### 2.2. Design and Fabrication of the 3D Printed Skull Cap

**Figure 3:**
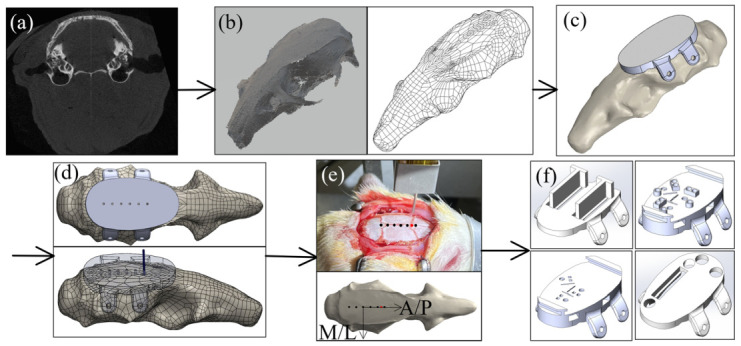
Design of the 3D printed skull cap and alignment with the anatomical feature: (**a**) CT-scan of the rat skull, (**b**) 3D reconstruction of the rat skull geometry based on CT scan, (**c**) design of a solid base cap geometry, (**d**) development of an anatomical alignment cap for bregma alignment test, (**e**) experimental alignment between the cap design and the rat bregma location, and (**f**) various skull cap designs with custom recording locations based on the universal bregma location.

To eliminate the labor-intensive manual alignment and temporary fixation of each MEA during the multi-region surgery, this study used a digitally designed and 3D printed skull cap. A computed tomography (CT) scan of the rat skull (Figure 3a) was first conducted using a microCT system (Skyscan 1176 by Bruker Corporation, Billerica, MA, USA) with 35 μm resolution. ImageJ was used to process the microCT images and to reconstruct the three-dimensional skull geometry (Figure 3b). The skull geometry was then imported into a computer-aided design (CAD) software (SolidWorks by Dassault Systems Inc., Waltham, MA, USA) for further designs. For a basic solid cap design (Figure 3c), the bottom of the cap matched the top surface of the skull and the top of the cap was designed to be a flat surface as the implantation apparatus platform. Four flaps were added on two sides of the skull for the fixation screw installation.

To evaluate the possibility of using the skull cap design based on a single Sprague-Dawley’s CT scan on different Sprague Dawley rats, a test was conducted aiming to quantify the variation of the bregma location among rats and to create a universal reference point on the headcap design to serve as the bregma reference location. Based on the geometry from the CT scan of a single rat skull, a stereolithography (SLA) 3D printer was used to create an alignment test headcap with six (6) 0.5 mm diameter through-holes separated by 2.2 mm along the anterior/posterior (A/P) direction (Figure 3d). This reference headcap was attached with screws onto Sprague Dawley rats (n = 6) varying in age, size, and body weight (Table 1) (surgical steps to be elaborated in Section 2.4). On each rat, a 0.5 mm male metal pin header (Model a18040700ux0248 by Uxcell, Hong Kong, China) was lowered into each reference hole on the cap, and the stereotaxic coordinates were documented. Using the fourth anterior dot as the origin, these dots formed a coordinate system based on the attached cap with the A/P direction along the dotted line and medial/lateral (M/L) direction perpendicular to it (Figure 3e). Without moving the animal’s position on the stereotax, the pin header was lifted above the animal, and the headcap was removed. The pin header was then lowered back down to the true bregma position on the animal’s skull, and the stereotaxic coordinates were again documented. The stereotaxic coordinates were used to calculate the position of the true bregma location on the skull for each animal corresponding to the A/P-M/L coordinate system built based on the skull cap design (Figure 3e). To evaluate the repeatability of using the general skull cap design geometry as a universal bregma point, both the standard error and the 95% confidence ellipse were calculated based on the rat bregma data (n = 6), as in Table 1, and the results are elaborated in the result section (Section 3.1). Based on the results, the point on the skull cap corresponding to the average bregma location was set as the universal headcap bregma point. This universal point could then be used as the reference for all implant target sites for all headcaps. Examples of the custom recording location designs, based on the universal bregma are shown in Figure 3f.

**Figure 4:**
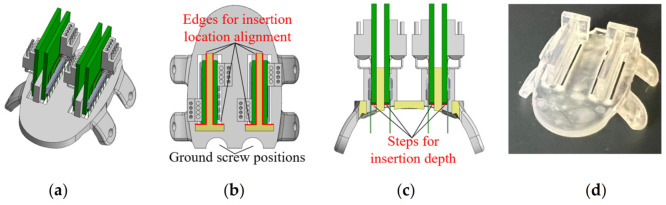
The custom skull cap used in this study: (**a**) design overview with four MEAs, (**b**) top and (**c**) front cross-sectional view of the cap showing the position alignment feature designs, and (**d**) picture of the 3D printed skull cap.

For the proof-of-concept animal surgery in this study, the features to be supported and/or guided by the custom skull cap, included four (4) microwire MEAs and two ground screws. For each MEA, the skull should provide the accurate positions and orientations along the medial/lateral and anterior/posterior directions as well as guidance and stop features along the implantation direction. As shown in Figure 4, based on the bregma aligned skull cap, the custom skull cap design introduced two half-circle openings on the back for the ground screw positioning (Figure 4b). A rectangular-shaped opening was cut at the insertion location of each MEA. Two T-shaped structures were created next to the openings so that during the insertion, the back and side edges of the MEA PCB could slide firmly against the corresponding support structure edges (highlighted in red in Figure 4b) without the need for a manual stereotactic alignment along each direction. Within the insertion opening of each MEA, a thin (0.25 mm thickness) step was created as the insertion depth indicator, as highlighted in red in Figure 4c. At the end of each MEA’s insertion, the PCB could sit on the 3D printed step, free of the extra support needed until the structure is fixed with dental cement. Note that the exact location of the step (insertion stopper) was determined by the targeted brain structure depth, the microwire overhang length from the MEA PCB, and the skull cap thickness. All caps in this study were printed using the same SLA 3D printer (Form 3B by Formlabs Inc., Somerville, MA, USA) and material (Clear Resin V4 by Formlabs Inc., Somerville, MA, USA) at a 25 µm resolution setting. The post-processing was conducted for the optimal part accuracy and performance (Form Wash and Form Cure by Formlabs Inc., Somerville, MA, USA).

As shown in Figure 3f, based on the specific experimental needs, the thickness and overall size of the cap could be adjusted, accordingly. If it is preferred to have the cap fulfill the craniotomy openings in the skull and rest against the brain membranes upon attachment, a 1.5 mm offset at the skull cap bottom was found in our preliminary study to fit most rats [32]. The support features and guidance openings could be designed on top of the cap, inside the cap, or offset beneath the cap bottom, based on the unique requirements of each experiment. This study presented an anatomical alignment with regard to a rat’s bregma, a similar methodology could be applied to various species and anatomical structures.

### 2.3. Benchtop Fabrication of Microwire-Based MEA

**Figure 5:**
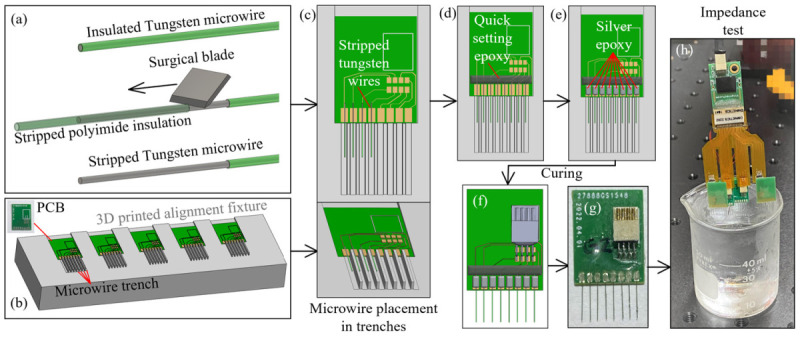
Fabrication process of the microwire-based MEA: (**a**) microwire insulation layer stripping with a surgical blade, (**b**) 3D printed fixture for the microwire alignment, (**c**) microwire placement along the 3D printed trenches, (**d**) epoxy fixation microwires, (**e**) microwire electrical connection with silver epoxy, (**f**,**g**) connector soldering, and (**h**) impedance test.

To fabricate the microwire-based MEAs used in this study, 50 µm diameter tungsten microwires with polyimide insulation (CFW2029287 by California Fine Wires Co., Grover Beach, CA, USA) were used as the raw material. The wires were hand-cut with fine surgical scissors (Artman Instruments, Kennesaw, GA, USA) into 6 mm long segments and a 2.2 mm long insulation layer was stripped from each segment by a razor blade (Figure 5a). To align the multiple microwires with the gold pads on the MEA PCB, an alignment fixture was designed and 3D printed (Figure 5b). The fixture had seven (7) evenly spaced ridges, forming eight (8) trenches whose centerlines coincident with the eight PCB gold pads (Figure 5c). The width of each trench (0.6 mm in this study) was chosen as a compromise between the wire placement difficulty and the parallel misalignment tolerance between the wires in an array. The MEA PCB was first placed in the designed opening to align the gold pads with the trenches. The stripped tungsten microwire was then placed in the middle of each trench with the stripped section laid over the gold pad (1.6 by 0.8 mm in size). The stripped insulation edge was aligned with the PCB edge so that an exact 3.8 mm overhang of each wire could be achieved. This overhang length, combined with the rest step in the skull cap design for the PCB and the removed skull cap thickness, would yield a desired 1.6 mm insertion depth into the rat brain as the targeted recording location (Figure 5c). These wire handling and skull cap design parameters can be adjusted to change the insertion depth. Once all of the wires are aligned in the trenches, about 0.5 mm of the stripped tungsten wires are extruded, beyond the top of the gold pad (Figure 5c). A small portion of the quick setting epoxy (ClearWeld by J-B Weld Company, Sulphur Springs, TX, USA) was applied on this stripped wire section, as in Figure 5d. This epoxy strip, along the back side of the gold pads, not only held all microwires firmly in place on the PCB, but also created insulation for any exposed tungsten tips.

Besides handling and placement, the soldering of the microwires was another labor-intensive and time-consuming step during the MEA fabrication. Worse yet, some microwire materials, such as stainless steel and tungsten, had nonoptimal solderability and would usually require extra steps of surface cleaning, pre-plating, pre-tinning, or specialized soldering [24,33,34]. To overcome these challenges, the conductive silver epoxy (EPO-TEK H20E by Epoxy Technology Inc., Billerica, MA, USA) was applied using a sewing needle to create the electrical connections between the microwires and the PCB gold pads (Figure 5e). The PCB with wires attached was heated in an oven at 150 °C for one hour for the silver epoxy curing. The standard (non-silver) epoxy is then used to coat and insulate the proximal ends of the wires and pads to prevent any crosstalk. The eight-pin connector (A79613-001 by Omnetics Connector Corporation, Minneapolis, MN, USA) was then soldered onto the rigid PCB board to finish the MEA fabrication process (Figure 5f,g). Prior to the implantation, each MEA went through an impedance test with its tips submerged into 1× phosphate-buffered saline (PBS), as shown in Figure 5h. The wire impedances were tested, using a 32-channel headstage (RHD2132 by Intan Technologies, Los Angeles, CA, USA) connected to a USB interface board (RHD2000 by Intan Technologies, Los Angeles, CA, USA). All of the implanted MEAs had wire impedances of 20–200 kΩ measured at 1 kHz.

This modular assembly approach, the 3D printed alignment fixture, and the usage of silver epoxy could significantly lower the time and effort for the MEA fabrication. For an inexperienced operator to build four 8-channel MEAs, it took about 3–4 h, including about 1.5 h of wire trimming, 1.5 h of wire alignment/fixation and connector soldering, and 1 h idle time for the silver epoxy curing. With an experienced operator, the entire process could be completed within 2–2.5 h including the 1 h oven curing time. This stands in significant contrast to the often expensive commercial solutions.

To generalize this benchtop MEA assembly approach, depending on the fabrication resources available, the 3D printed alignment fixture with ridges could also be made by precision milling of metal blocks or chemical etching on silicon, to fulfill the different MEA pitch size needs. If the raw microwire material was un-insulated when initially obtained, an extra insulation step (e.g., conformal coating) would need to be added at the beginning or after the assembly. If the impedance test yielded a higher value than preferred, an additional conductive coating (e.g., PEDOT:pTS, platinum-iridium) could be applied through electroplating [35]. MEAs with microwires in equal length were demonstrated in this study. The alignment fixture method could be easily applied to more complicated linear MEA configurations with various electrode lengths in an array.

### 2.4. Skull Cap Based MEA Implantation and Electrophysiological Recording

**Figure 6:**
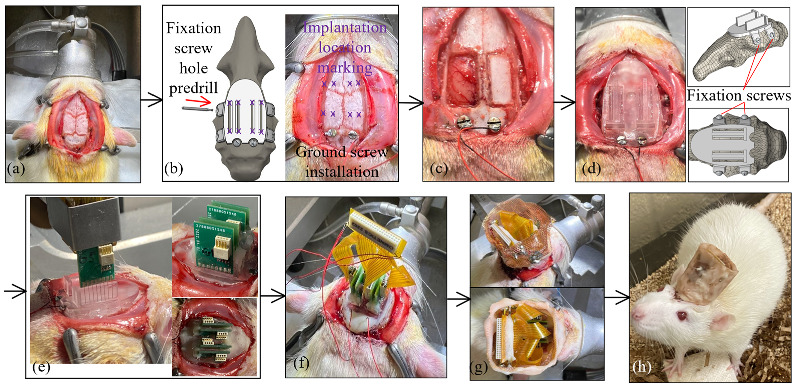
Surgical procedure for the skull cap installation and the cap-based MEA implantation: (**a**) incision of the scalp, (**b**) positioning with the template cap, (**c**) craniotomy, (**d**) installation of the custom skull cap, (**e**) implantation of the MEA, (**f**) electrical connection with the flexible adapter, (**g**) Faraday cage installation and dental acrylic sealing, and (**h**) animal after surgery.

Figure 6 presents the surgical procedures for implanting the fabricated MEAs at pre-printed locations through the custom skull cap into a certain depth in the rat brain. All animal experiments in this study were carried out in accordance with the University of Michigan Institutional Animal Care and Use Committee (protocol number PRO00009818 approved for 13 July 2020–13 July 2023). The electrode insertions were performed on 3–6 months old Sprague Dawley rats (Charles River). All of the animals were housed on a normal light dark cycle (lights on 7 a.m.–7 p.m.) in a standard enriched environment and fed ad libitum. The rats were anesthetized with isoflurane, placed in a stereotaxic apparatus, and given a subcutaneous injection of Carprofen 5 mg/kg for analgesia, as well as methylprednisolone 30 mg/kg intraperitoneally, to limit brain swelling. A local anesthetic, Bupivacaine 1 mg/kg, was administered subcutaneously to the scalp before the surgical incision. An incision was made on the scalp along the midline of the skull with a sterile scalpel blade to expose the skull (Figure 6a). Prior to surgery, a template skull cap was designed and printed (Figure 6b). It had the same geometry as the final skull cap as in Figure 4d, except that it did not have the T-shaped support structure for an easier reach of the insertion opening bottoms. This template cap was placed onto the skull surface and adjusted around, for the best fit. It served as the surgical coordinate template and eliminated the needs for stereotactic measurements of all skull operation locations. A dental drill (MH-170 by Foredom, Bethel, CT, USA) with the burr bit size of 0.9 mm diameter was used to pre-drill four fixation screw holes on the side of the skull for the later anchoring of the headcap to the skull. Two stainless steel screws (00–90, 3/32″) were driven through the skull over the cerebellum to serve as ground and reference. The burr was also inserted through the four MEA implantation openings and made marks on the skull as the craniotomy reference locations (Figure 6b). Following the template cap removal, two craniotomy windows were cut on the skull followed by durotomy of the opened regions (Figure 6c). The craniotomy windows were filled with petroleum jelly, and the custom 3D printed skull cap, as shown in Figure 4d, was then attached to the skull by tapping four fixation screws (00–90, 1/8″) through the pre-drilled holes (Figure 6d). During the attachment, both the fixation screw holes and the two installed ground screws served as the reference points to ensure the proper alignment of the cap. The dental acrylic (UNIFAST Trad by GC America Inc., Alsip, IL, USA) was used to seal the headcap-skull junction along the cap perimeter.

The MEA assembled, as elaborated in Section 2.3, was gripped by a stereotactic arm (Model 1771 by David Kopf Instruments, Tujunga, CA, USA) and was aligned against the T-shaped structure on the custom skull cap, as shown in Figure 6e. The MEA was then inserted until the PCB was lowered into contact with the insertion depth indicator step, as in Figure 4c. During the implantation, the stereotactic arm was used only as a manipulator and no manual quantitative distance measurement was needed. Following the implantation, as in Figure 6e, each MEA was anchored onto the skull cap with a dental acrylic (UNIFAST Trad by GC America Inc., Alsip, IL, USA). When all four MEAs were secured, the flexible adapter PCB was used to connect four eight-pin Omnetics connectors on the MEAs to a 36-position connector for the headstage (Figure 6f). To protect the recording apparatus from the external electromagnetic waves, a Faraday cage was built around the PCBs with a copper mesh and the ground screw wires were connected to the cage. The cage base was cemented to the perimeter of the headcap, and the cage body was covered with a layer of dental acrylic, as shown in Figure 6g,h. The rats recovered for seven days, then the electrophysiological recordings were performed in the rats’ home-cage, using a 32-channel headstage (Intan Technologies RHD2132) connected to a USB interface board (Intan Technologies RHD2000) and sampled at 20 kHz.

## 3. Results

### 3.1. Bregma Localization

**Figure 7:**
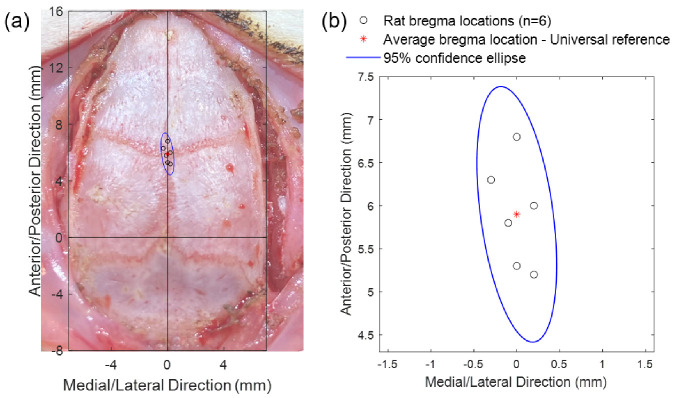
Headcap bregma reference point compared to the true bregma of each animal: (**a**) Plot of the difference between the true bregma locations of six animals (circles) and the averaged skull cap bregma reference point (asterisk) overlayed onto the picture of the typical dorsal skull (aligned at bregma) to emphasize the precision and accuracy of the universal headcap bregma. The skull surface measures approximately 14 × 24 mm. (**b**) Close-up view of the bregma location variance and 95% confidence ellipse.

The coordinates of the true bregma positions of six Sprague Dawley rats in the A/P–M/L coordinate system, with the fourth anterior hole in the template cap as origin, are listed in Table 1. The average position of the six true bregma points was obtained by taking the average values of the six A/P and M/L coordinates, respectively, and the result was (0.0, 5.9) (unit: mm). This average point, as highlighted by the red asterisk in Figure 7, was used as the universal bregma reference point for the skull cap design. As seen in Figure 7b, the full range of the error between the true bregma locations and this universal headcap bregma (n = 6) was within +/− 1.0 mm along the A/P axis and +/− 0.3 mm along the M/L axis (A/P axis standard error of the mean = 0.25 mm, M/L axis standard error of the mean = 0.07 mm, n = 6; as in Figure 7). The 95% confidence ellipse is also shown in Figure 7 with the size within ± 1.5 mm along the A/P direction and ± 0.5 mm along the M/L direction. The precision of the headcap positioning (using bregma as the reference) across six animals of varying age, size, and weight (Table 1) demonstrates the universality of the headcap design. Even though it would be the most accurate to take CT scan of each individual rat and design custom skull cap accordingly, the accuracy of this universal bregma point shows the feasibility to fabricate the custom target openings on the cap prior to surgery, without CT scan, and be able to reliably implant our arrays without stereotactic guidance.

### 3.2. Brain Recording Results

Four MEAs containing eight wires each, were inserted through the headcap, as detailed above. The 32 wires formed a rectangular grid spanning 7 mm along the anterior/posterior axis and 10 mm along the medial/lateral axis to allow for the broad sampling of the cortical activity. All wires were inserted between the coronal and lambdoid skull sutures, to a depth of 1.6 mm. The high-resolution recordings were collected at 20 kHz to obtain the local field potential and single unit signals.

**Figure 8:**
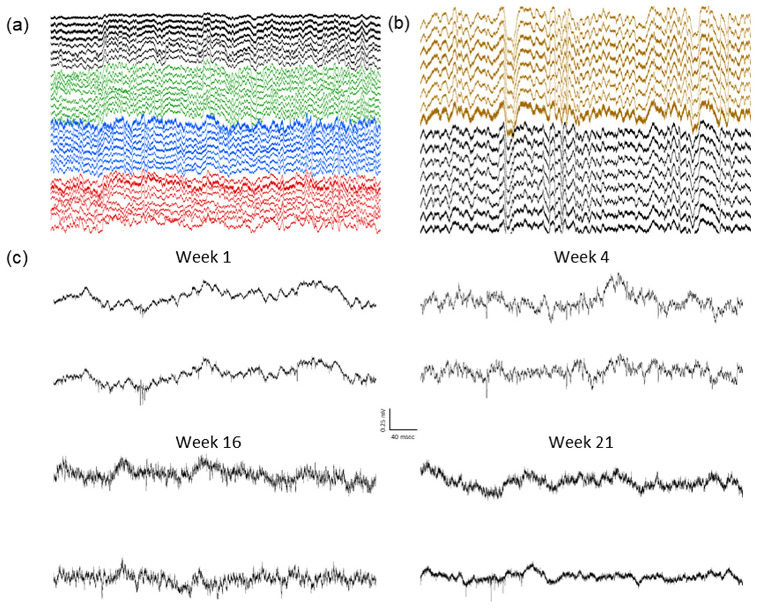
Example recordings from an animal with four eight-wire array implants: (**a**) Example two seconds of 32-channel recording. Individual arrays are highlighted in different colors. (**b**) Example four seconds showing delta oscillations (0.5–4 Hz) characteristic of normal physiology during NREM sleep. (**c**) Spiking activity in two example channels from the same animal over a five-month period.

The local field potentials (LFP) from 32 channels were recorded over many weeks, during the home-cage sessions, each lasting 2–6 h (Figure 8a). The presence of delta oscillations (0.5–4 Hz) during non-rapid-eye-movement (NREM) sleep indicate a normal physiology and successful cortical LFP recordings (Figure 8b). The single units were also observed in the multiple channels for more than five months (Figure 8c), indicating stable high-resolution recording capabilities. Despite removing a large portion of the dorsal calvarium, none of the animals showed any behavioral changes or deficits, and there were no signs of infection or other complications. The signals could be maintained for multiple months, in a manner comparable with more traditional surgery methods.

## 4. Discussion

As seen in the results section, the top portions of the skull can be replaced by printed parts without compromising animal health, behavior, or recording quality. We’ve also shown that our cap can be used to insert many wires that yield stable and high-resolution recordings over multiple months. Our CT-aided design conforms well to the skulls of Sprague Dawley rats of varying size, age, and weight, and creates a minimal bregma alignment error. For higher resolution alignment needs, CT head scans (bone) and T1 magnetic resonance imaging (MRI) scan (brain structure) could be co-registered to directly correlate the custom skull cap design with targeted brain structures [32].

In-vivo rat brain recording results from the proof-of-concept study showed the feasibility of the developed design, fabrication, and implantation methodologies to deliver high-quality chronic neural recordings. Most of the methodologies showcased in this proof-of-concept study could be generalized to the electrophysiological recordings and stimulations on the different species, with various devices and configurations, thanks to the capability provided by the benchtop custom MEA fabrication process, CAD, and 3D printing. Because our caps are 3D-printable, they are affordable, widely accessible, and can also be tailored for custom recording locations for specific regions or more broadly for large-scale cortical recordings. This new implantation method could also accommodate other recording designs, including micro-drive manipulators, tetrodes, silicon probes, optogenetic fibers, and imaging windows. Each of these recording options can be planned prior to surgery to reduce the manual manipulation and implantation difficulty, resulting in surgeries that are more efficient and repeatable, and offering high-resolution and high-density and/or broad-scale sampling, and providing stable chronic recordings.

Given that brain dynamics involve multi-region coordinations, the ability to better study those dynamics will depend on the development of systems to implant complex arrays of probes that are not practically feasible with manual methods. Computer-based designs and the printing of surgery-enabling platforms is very likely to become increasingly commonplace as rodent brain surgeries become more complex. The developed system represents an important step towards the computer-aided rodent brain implantation to better study the brain and the disease models, from the perspective of broad-scale electrophysiologic dynamics.

This study has shown that the development of a new methodology, based on a computer-aided designed and 3D printed skull cap system for both MEA fabrication and rat brain surgery. This work reduces the labor and time for the creation and the surgical implantation of MEAs and allows laboratories to undertake the fabrication on their own, at low cost. The printed skull replacement with the ability to pre-shape and pre-form craniotomies and mounts for surgeries, provides a system that is convenient and effective now, but also is an important first step for future developments.

In the developed benchtop fabrication process, while largely improved, the microwire placement and alignment step still requires an extensive labor effort. Furthermore, the impedance result of the assembled MEAs depended upon the wire stripping/cut off and silver epoxy application quality. Future work to further improve the fabrication process will be focused on the machine-based assembly of the microwire MEAs, including the automatic wire feeding and the alignment and machine-controlled epoxy dispensing. For the 3D printed skull cap, future studies will focus on the design considerations to further minimize the bregma location error, along the A/P direction and expansion of the skull cap concept into other rodent species and even primates.

## 5. Conclusions

A new methodology for laboratories to fabricate a low-cost custom microwire-based MEAs for the multiple brain regions’ implantation, based on a computer-aided and designed 3D printed skull cap platform, has been developed.

The skull cap design, based on the CT-scan of a single Sprague Dawley rat, fits the skull of varying sizes, ages, and weight Sprague Dawley rats (n = 6), with standard error of 0.25 mm and 0.07 mm along the A/P and M/L directions, respectively, indicating the feasibility to identify the implantation locations on a general skull cap design, without an animal-specific CT scan and be able to reliably implant the arrays without stereotactic guidance.

Based on the proof-of-concept design, the prototype, and the animal recording study, the benchtop fabricated MEAs implanted through the 3D printed skull cap, were shown to be capable of recording spiking activities over multiple months.

The design, fabrication, and implantation methodologies elaborated in this study could be duplicated, reconfigured, and/or generalized to various devices and configurations on different species, to enable low-cost highly custom multi-region recording/stimulation studies.

## Figures and Tables

**Table 1:** Age and weight of all rats used in the bregma localization and error trials.

Rat	Age (Weeks)	Weight (g)	Animal Bregma A/P Coordinate (mm)	Animal Bregma M/L Coordinate (mm)
1	6	360	5.2	0.2
2	6	360	5.3	0.0
3	4	266	5.8	−0.1
4	4	265	6.0	0.2
5	5	605	6.8	0.0
6	6	405	6.3	−0.3

## Data Availability

The data presented in this study are available on request from the corresponding author.
